# Airway smooth muscle chemokine receptor expression and function in asthma

**DOI:** 10.1111/j.1365-2222.2009.03310.x

**Published:** 2009-11

**Authors:** R Saunders, A Sutcliffe, D Kaur, S Siddiqui, F Hollins, A Wardlaw, P Bradding, C Brightling

**Affiliations:** Department of Infection, Immunity and Inflammation, Institute for Lung Health, University of Leicester>Leicester, UK

**Keywords:** asthma, airway smooth muscle, chemokine receptors, wound healing

## Abstract

**Background:**

Chemokine receptors play an important role in cell migration and wound repair. In asthma, CCR3 and 7 are expressed by airway smooth muscle (ASM) and CCR7 has been implicated in the development of ASM hyperplasia. The expression profile of other chemokine receptors by ASM and their function needs to be further explored.

**Objective:**

We sought to investigate ASM chemokine receptor expression and function in asthma.

**Methods:**

ASM cells were derived from 17 subjects with asthma and 36 non-asthmatic controls. ASM chemokine receptor expression was assessed by flow cytometry and immunofluorescence. The function of chemokine receptors expressed by more than 10% of ASM cells was investigated by intracellular calcium measurements, chemotaxis, wound healing, proliferation and survival assays.

**Results:**

In addition to CCR3 and 7, CXCR1, 3 and 4 were highly expressed by ASM. These CXC chemokine receptors were functional with an increase in intracellular calcium following ligand activation and promotion of wound healing [CXCL10 (100 ng/mL) 34 ± 2 cells/high-powered field (hpf) vs. control 29 ± 1; *P*=0.03; *n*=8]. Spontaneous wound healing was inhibited by CXCR3 neutralizing antibody (mean difference 7 ± 3 cells/hpf; *P*=0.03; *n*=3). CXC chemokine receptor activation did not modulate ASM chemotaxis, proliferation or survival. No differences in chemokine receptor expression or function were observed between ASM cells derived from asthmatic or non-asthmatic donors.

**Conclusions:**

Our findings suggest that the chemokine receptors CXCR1, 3 and 4 modulate some aspects of ASM function but their importance in asthma is uncertain.

## Introduction

Asthma is characterized by typical symptoms, airway hyperresponsiveness (AHR) and variable airflow obstruction, which can become fixed in severe disease. In addition, there is associated airway inflammation, which is usually eosinophilic, together with features of tissue repair known as remodelling [1]. Airway remodelling in asthma encompasses several structural changes in the airway wall including reticular lamina and basement membrane thickening and increased airway smooth muscle (ASM) mass [2, 3]. This latter feature is due to a combination of both ASM hyperplasia [4] and hypertrophy, which increases with disease severity and is associated with fixed airflow obstruction [2, 5].

The cause of ASM hyperplasia in asthma is unknown and is often attributed to increased proliferation. Indeed proliferation is increased in *ex vivo* asthmatic ASM in some studies [6, 7] but not others [8, 9], and several reports have been unable to demonstrate increased ASM proliferation *in vivo* [4, 5, 10]. An alternative explanation is that ASM or its progenitors migrate to the ASM bundle. It is likely that this recruitment will require a chemotactic signal arising from the ASM. The C–C and C–X–C chemokines, in particular, are attractive candidates as ASM chemoattractants. These ubiquitous, structurally related peptides mediate the chemotaxis of many cell types [11, 12]; play a key role in wound repair [13] and in regulating cell survival and proliferation [14–17]. In asthma, ASM contributes to the secretion of pro-inflammatory mediators and is an important source of chemokines [18]. However, in contrast to the extensive literature on ASM-derived chemokines there is a paucity of data describing the expression and function of ASM chemokine receptors. To date only CCR1, 3 and 7, and CXCR1 and 2 have been reported, but the relative contribution of these and possibly other chemokine receptors to ASM function in asthma is uncertain [19–23].

We hypothesized that: (i) ASM cells express a range of chemokine receptors, (ii) the pattern of expression is different in subjects with and without asthma, (iii) the chemokine receptors expressed are functional; promote ASM migration and repair, and modulate cell survival and proliferation. To test our hypothesis, we examined chemokine receptor expression and function using a variety of techniques in health and disease.

## Materials and methods

### Subjects

Asthmatic subjects and non-asthmatic controls were recruited from Leicester, UK. Subjects with asthma had a consistent history and objective evidence of asthma, as indicated by one or more of the following: (1) methacholine AHR (PC_20_FEV_1_<8 mg/mL); (2) >15% improvement in FEV_1_ 15 min after administration of 200 μg of inhaled salbutamol; or (3) >20% of maximum within-day amplitude from twice daily peak expiratory flow measurements over 14 days. The study was approved by the Leicestershire Ethics Committees and all patients gave their written informed consent.

### Airway smooth muscle and mast cell isolation and culture

Pure ASM bundles in bronchial biopsies obtained from fibreoptic bronchoscopy (*n*=21, 17 asthmatic subjects, four non-asthmatic) and additional airways isolated from lung resection (*n*=32) were dissected free of surrounding tissue. Primary ASM was cultured and characterized as previously described [24]. The clinical characteristics of the ASM donors are as shown in Table 1.

**Table 1:** Clinical characteristics [mean (SEM)]

	Asthma	Controls
Number	17	36
Gender (M/F)	8/9	25/11
Age (years)	53 (4)	65 (3)
FEV_1_ (L)	2.3 (0.2)	2.1 (0.1)
FEV_1_% predicted	77 (6)	81 (5)
FEV_1_/FVC (%)	67 (3)	74 (3)

FEV_1_, forced expiratory volume in 1 s.

Human lung mast cells (HLMC) were isolated and cultured from non-asthmatic lung (*n*=3) as previously described [25].

### Chemokine receptor protein expression

#### Flow cytometry

ASM were stained with antibodies to the following chemokine receptors: mouse mAb CCR1, 2, 3, 4, 5 and 6, CXCR1, 2, 3, 4, 5 and 6 (R&D Systems, Abingdon, Oxfordshire, UK), and CCR7, 9, and 10 (gift from Millenium, Cambridge, MA, USA); rabbit polyclonal antibodies CCR8 (AMS Biotechnology, Abingdon, Oxfordshire, UK) and CX_3_CR1 (Chemicon, Hampshire, UK). These were indirectly labelled with fluorescein isothiocyanate (FITC), and appropriate isotype controls were performed (mouse mAb IgG1, IgG2a, IgG2b or mAb rabbit IgG, Dako, Stockport, UK), then analysed using single colour flow cytometry on a FACScan (BD Bioscience, Oxford, UK). Chemokine receptors with >10% expression were further examined, excluding CCR3 and 7 as we have previously described the findings for these receptors [21, 22].

#### Immunofluorescence

ASM were grown to confluence on chamber slides and serum deprived for 24 h. The cells were labelled with the appropriate mAb or isotype control as used for flow cytometry, and indirectly labelled with FITC. Cells were counterstained with 4′,6′-diamidino-2 phenylindole (DAPI, Sigma, Gillingham, Dorset, UK).

### Chemokine receptor mRNA expression

ASM chemokine receptor mRNA expression was assessed in ASM cells from asthmatic (*n*=3) and non-asthmatic donors (*n*=3) following incubation with poly(inosinic:cytidylic) acid (poly(I:C), Sigma) vs. poly(deoxyinosinic–deoxycytidylic) acid (poly(dI:dC), Sigma) control at 2.5 ng/mL for 4 h and following incubation with supernatants from IgE/anti-IgE activated HLMC (10 × 10^6^ cells pooled from three donors) for 6 and 24 h. The proportion of HLMC : ASM cells was 1 : 4. RNA expression levels of chemokine receptors extracted from the ASM was examined using the Human Genome U133A probe array (GeneChip, Affymetrix, Santa Clara, CA, USA). RNA was prepared and analysed as described [26]. Hybridized biotinylated cRNA was stained with streptavidin–phycoerythrin (Molecular Probes, Eugene, OR, USA), scanned with a HP Gene Array Scanner (Affymetrix), and data analysed using the GeneChip Analysis Suite 4.0/Operating System (Affymetrix) as described Bradding et al. [26].

### Functional assessment of airway smooth muscle chemokine receptors

#### Calcium imaging

Changes in cytosolic Ca^2+^ concentration ([Ca^2+^]_i_) in ASM cells in response to appropriate ligands (100 ng/mL) and bradykinin (1 ng/mL) as a positive control were measured by ratiometric imaging on FURA-2-loaded cells using Openlab software (Improvision, Coventry, UK). This was converted to [Ca^2+^]_i_ using a calibration kit (Invitrogen Molecular Probes, Paisley, Scotland, UK). Cells were considered to have responded to a ligand if the increase in [Ca^2+^]_i_ exceeded the mean+2 standard deviations of the baseline.

#### Wound-healing assay

ASM cells were seeded onto eight rectangular well plates coated with 10 μg/mL fibronectin at a density of 0.25 × 10^6^ cells/well, allowed to adhere overnight, then serum deprived in insulin/transferrin/sodium selenite (ITS) (ITSx3; Sigma) media for 24 h before experimentation. Wounds were introduced using a sterile 10 μL pipette tip. The number of cells that moved into the wound in the presence of chemokines (25–300 ng/mL) or ITS control media in the presence or absence of the appropriate neutralizing antibody or isotype control (R&D) over 6 h were counted by a blinded observer [22].

#### Chemotaxis assay

We used a validated chemotaxis assay [22]. In brief, ASM cells were seeded as per the wound-healing assay. Cells were removed by scraping between the top of the well and a line predrawn across the width of the well, on the underside of the plate, 22 mm from the bottom of the well. Cell debris was removed by washing with ITS media. Blotting paper (25 mm × 6 mm; Sigma) was then placed along the upper edge of the well, secured in place using silicon grease. Chemokines (12.5–200 ng; R&D) or ITS control media was impregnated onto blotting paper from which it diffused into the media. The number of cells that moved towards the resultant chemokine concentration gradient were enumerated after 6 h by a blinded observer.

#### Cell metabolic activity

ASM cell metabolic activity was assessed using the 3-(4,5-dimethylthiazol-2-yl)-5-(3-carboxymethoxyphenyl)-2-(4-sulfophenyl)-2H tetrazolium inner salt (MTS) assay, according to the manufacturer's instructions (Promega, Southampton, UK), following incubation with chemokines in 10% fetal bovine serum (FBS) media (12.5–100 ng/mL) or ITS media (100 ng/mL) or in the presence of appropriate neutralizing antibodies and isotype controls (R&D) for 24 and 96 h.

#### Proliferation and survival

ASM proliferation was assessed using the CellTrace CFSE Cell Proliferation Kit according to the manufacturer's instructions (Invitrogen Molecular Probes). Cells treated with 50 μg/mL mitomycin C (Sigma) for 3 h to mitotically arrest cells at the parent population, before re-addition of 10% FBS media, were cultured in parallel to cells exposed to 10% FBS media ± 100 ng/mL of chemokines for 96 h.

The percentage of apoptotic ASM cells exposed to 10% FBS media ± 100 ng/mL of chemokines was identified by DAPI staining of cell nuclei after 24 and 96 h, and by staining with FITC-conjugated Annexin V (1 μL/200 μL binding buffer, BD Bioscience) ± propidium iodide (PI, 0.5 μg/mL, BD Bioscience) after 96 h, before analysis on a FACScan (BD Bioscience).

### Statistical analysis

Statistical analysis was performed using GraphPad Prism 4 (GraphPad software, San Diego, CA, USA). Data are presented as mean ± SEM. Data was analysed by anova across groups and *t*-tests between groups. Differences were considered significant when *P*<0.05.

## Results

### Chemokine receptor expression by airway smooth muscle

**Fig. 1:**
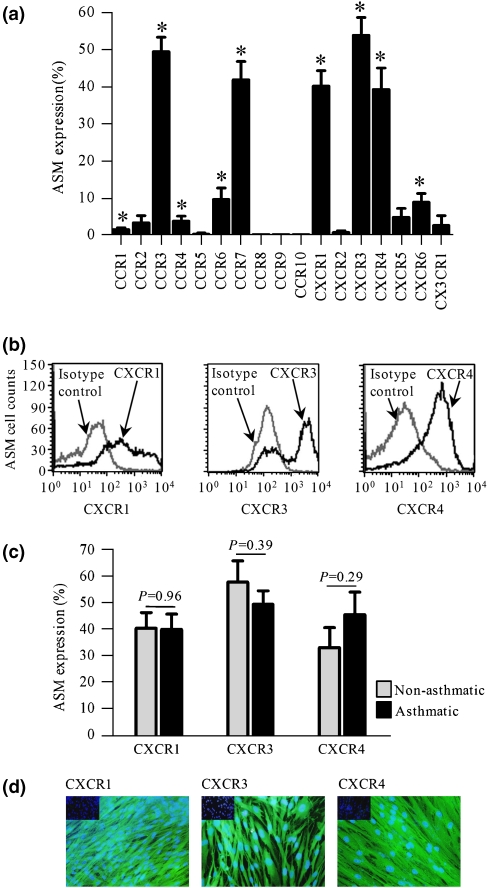
Chemokine receptor expression by primary cultured airway smooth muscle (ASM) cells. (a) Chemokine receptor expression by ASM cells assessed by flow cytometry (*n*=6–21, ^*^P<0.05 compared with appropriate isotype control). (b) CXCR1, 3 and 4 expression by ASM cells. Fluorescent histograms (black lines), plotted with corresponding isotype controls (grey line). (c) Percentage expression in asthmatic (*n*=10) and non-asthmatic (*n*=11) ASM cells. (d) Immunofluorescence (nuclei stained blue, chemokine receptor stained green; inset: isotype control).

We examined the expression of chemokine receptors by ASM using flow cytometry. ASM expression was statistically significant compared with isotype control for CCR1, 3, 4, 6, 7, CXCR1, 3, 4 and 6, and was also >10% for CCR3, 7, CXCR1, 3 and 4 (Fig. 1a). We have previously reported expression of CCR3 and 7 by ASM [21, 22]. Example flow cytometry histograms for CXCR1, 3 and 4 are as shown in Fig. 1b. The proportion of primary cultured ASM cells that expressed cell surface CXCR1, 3 and 4 was not different between those subjects with or without asthma (Fig. 1c). Expression of these chemokine receptors was also confirmed by immunofluorescence (Fig. 1d).

There was no difference in ASM chemokine receptor mRNA expression between subjects with and without asthma, in unstimulated ASM cells or following incubation with poly(I:C) or HLMC supernatants (data not shown).

### Airway smooth muscle CXCR1, 3 and 4 function

#### Calcium imaging

**Fig. 2:**
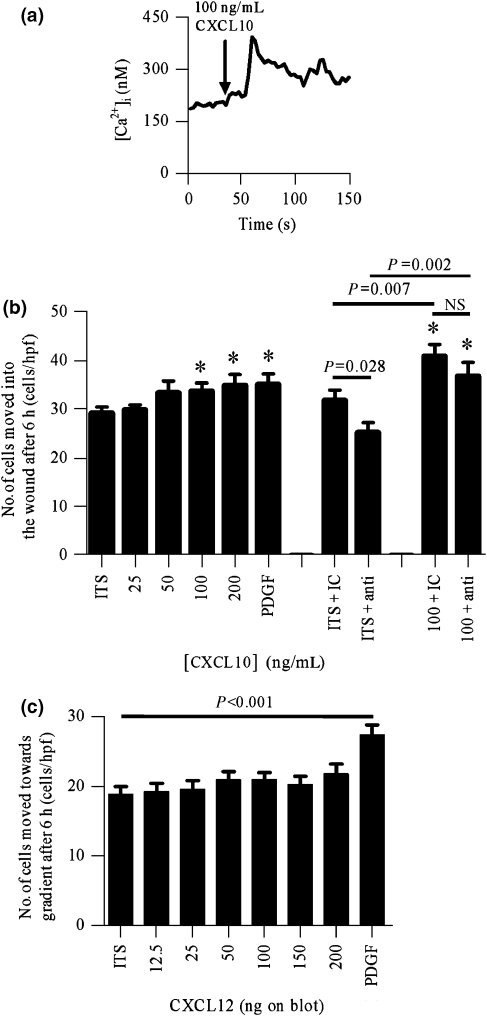
Chemokine receptor-mediated airway smooth muscle (ASM) wound healing and chemotaxis. (a) Representative trace illustrating the [Ca^2+^]_i_ elevation in ASM cells following addition of 100 ng/mL of CXCL10 (indicated by arrow). Similar responses were observed for CXCL8 and 12. (b) Increased wound healing was observed for CXCL8, 10 and 12. The ASM wound-healing response in the presence and absence of CXCL10 and a CXCR3 neutralizing antibody (anti, 15 μg/mL) or isotype control (IC) is shown (*n*=3–8, ^*^P<0.05 compared with ITS). (c) CXCL8, 10 and 12 did not promote chemotaxis. Example of ASM chemotaxis towards CXCL12 is as shown with PDGF (10 ng) as a positive control (*n*=6, comparisons made to ITS alone). Data are presented as mean ± SEM. ITS, insulin/transferrin/sodium selenite; PDGF, platelet-derived growth factor.

A transient mean increase in [Ca^2+^]_i_ was seen following activation of ASM with recombinant CXCL8, 9, 10, 11 or 12 (100 ng/mL) and bradykinin (1 ng/mL) (see Table 2). An example trace following activation with CXCL10 is as shown in Fig. 2a.

**Table 2:** ASM intracellular calcium response to activation by CXCR1, 3 and 4 ligands (100 ng/mL)

	CXCL8	CXCL9	CXCL10	CXCL11	CXCL12	Bradykinin
Cells (*n*)	25	19	26	23	26	51
Cells responding (%)	84	11	92	70	89	90
Δ[Ca^2+^]_i_ (nm) in responding cells	228 ± 44	98 ± 162	169 ± 64	136 ± 61	168 ± 79	255 ± 16

ASM, airway smooth muscle.

#### Wound healing

Recombinant CXCL8, 10 and 12 all promoted wound healing in a concentration-dependent manner. The chemokine-mediated wound healing was significant for CXCL8 at 50 ng/mL (34.6 ± 2.7 cells/high-powered field (hpf) vs. 27.6 ± 2.0 in control; *P*=0.04; *n*=4), for CXCL10 at 100 ng/mL and 200 ng/mL (33.8 ± 1.7 and 35.0 ± 2.2 cells/hpf vs. 29.3 ± 1.2 in control; *P*<0.05; *n*=8), and for CXCL12 at 200 ng/mL (40.6 ± 2.5 cells/hpf vs. 34.3 ± 1.6 in control; *P*=0.03; *n*=6). Data for the response to CXCL10 only is shown as the response to other chemokines was similar (Fig. 2b). The wound-healing response in the presence of ITS media alone was significantly reduced in the presence of a CXCR3 neutralizing antibody (mean difference 6.6 ± 2.9 cells/hpf; *P*=0.03; *n*=3; Fig. 2b), but not CXCR1 or 4 neutralizing antibodies (data not shown). Platelet-derived growth factor (PDGF) was included as a positive control. PDGF-mediated wound healing was significantly increased compared with control. For all experiments, combined PDGF-mediated wound healing was 38.9 ± 1.4 vs. control 32.8 ± 1.4 cells/hpf (11 donors, mean difference 6.1 ± 2.0 cells/hpf; *P*=0.002).

#### Chemotaxis

Recombinant CXCL8, 10 or 12 (12.5–200 ng) did not mediate dose-dependent ASM chemotaxis (*n*=4, *P*>0.05). Data for CXCL12 only is shown as the response to other chemokines was similar (Fig. 2c). As per the wound-healing assays, PDGF was included as a positive control for all chemotaxis assays and migration towards PDGF was significantly increased compared with the control [for all experiments combined PDGF-mediated migration (22.2 ± 1.1 cells/hpf [SEM]) vs. control (19.1 ± 0.8 cells/hpf [SEM]) (11 donors, mean difference 3.1 ± 1.3 cells/hpf; *P*=0.02)].

#### Metabolic activity

**Fig. 3:**
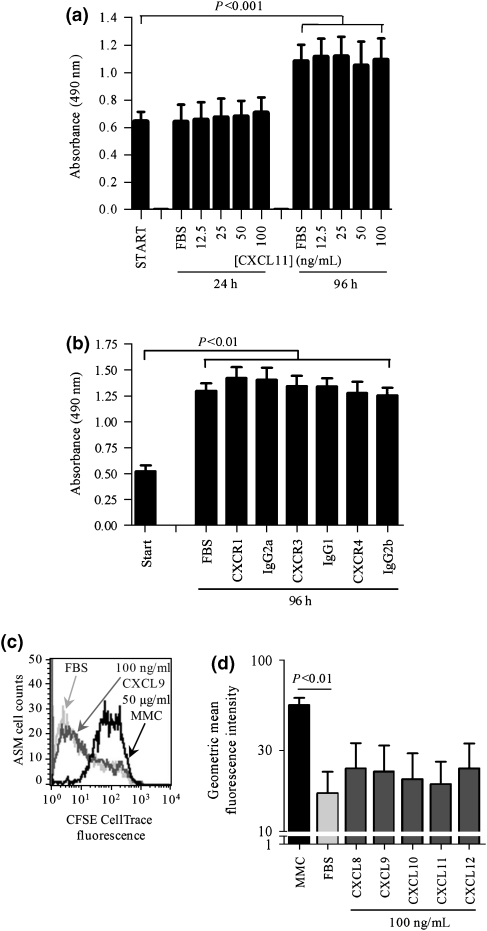
Activation of CXCR1, 3 or 4 has no effect on airway smooth muscle (ASM) cell metabolic activity or proliferation. Cell metabolic activity in the presence of (a) fetal bovine serum (FBS) media ± CXCL11 (*n*=6, data are presented as mean ± SEM) and (b) CXCR1, 3 and 4 neutralizing antibodies and appropriate isotype controls (*n*=4, data are presented as mean ± SEM), for 96 h. (c) A representative histogram illustrating CFSE fluorescence in ASM cells incubated with 50 μg/mL MMC (black line) or FBS media ± 100 ng/mL CXCL9 for 96 h (dark grey and light grey lines, respectively). (d) ASM cell proliferation was observed after 96 h, but was unaffected by incubation with CXCL8–12 (100 ng/mL) (*n*=6, data are presented as geometric mean ± SEM).

The absorbance by formazan seen at 490 nm in the MTS assay was increased in ASM after 96 h in both 10% FBS media and ITS media compared to ASM at 0 h. Recombinant CXCL8, 9, 10, 11 and 12 (12.5–100 ng/mL) had no effect on the MTS assay in the presence of FBS media (*n*=6, *P*>0.05), with no difference observed between ASM cells derived from non-asthmatic vs. asthmatic donors. Data for CXCL11 only is shown as the response to other chemokines was similar (Fig. 3a). In the presence of ITS media, recombinant CXCL8, 9, 10, 11 and 12 (100 ng/mL, *n*=4, *P*>0.05) had no effect on the absorbance measured at 490 nm (data not shown). Incubation with neutralizing antibodies to CXCR1, 3 and 4 had no effect on the MTS assay in the presence of FBS media compared with appropriate isotype controls (*n*=4, *P*>0.05, Fig. 3b).

#### Proliferation

Using the CellTrace CFSE Cell Proliferation assay, cell proliferation was seen after 96 h in the presence of 10% FBS media compared with MMC-treated cells (mean decrease in fluorescence intensity 38.2 ± 5.9, *P*<0.01, *n*=6, Figs 3c and d). This was unaffected by incubation with CXCL8–12 (100 ng/mL) (*n*=6; Fig. 3d), with no difference observed between ASM cells derived from non-asthmatic vs. asthmatic donors (data not shown).

#### Survival

**Fig. 4:**
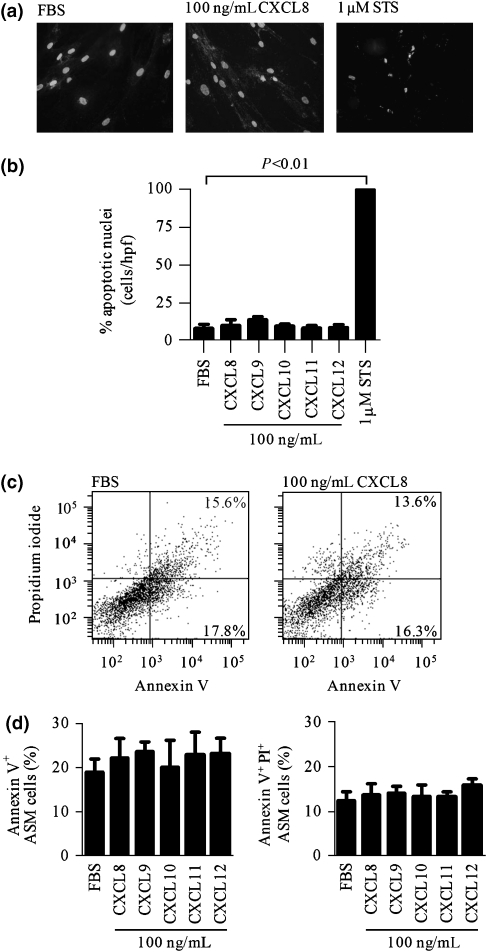
Activation of CXCR1, 3 or 4 has no effect on airway smooth muscle (ASM) cell survival. (a) Representative photographs showing DAPI staining of nuclear morphology in the presence of fetal bovine serum (FBS) media or 100 ng/mL CXCL8 for 96 h, or STS as a positive control. (b) Percentage of apoptotic ASM cells following culture with CXCL8–12 (100 ng/mL) or STS (1 μM). (c) Dot blots illustrating percentage of annexin V^+^/PI^−^ (lower right) and annexin V^+^/PI^+^ (upper right) ASM cells using two-colour flow cytometry in the presence of FBS media or 100 ng/mL CXCL8 after 96 h. (d) Percentage of annexin V^+^/PI^−^ and annexin V^+^/PI^+^ cells following culture with CXCL8–12 (100 ng/mL) (*n*=6, data are presented as mean ± SEM).

Following DAPI staining of ASM nuclei under control conditions, a low percentage of cells (6.8 ± 3.3%) showed nuclear condensation and fragmentation characteristic of apoptosis. The percentage of apoptotic cells was unaffected by incubation with CXCL8–12 (100 ng/mL) for 24 or 96 h (*P*>0.05, *n*=6). In marked contrast in the presence of staurosporine (STS, 1 μm, 20 h), a positive control, 99.7 ± 0.2% of ASM cells showed nuclear morphology characteristic of cells undergoing apoptosis (*P*<0.01; *n*=6) (Figs 4a and b).

The above data were confirmed using annexin V/PI staining of ASM cells. The percentage of annexin V^+^/PI^−^ (early apoptotic [27]) ASM cells was unaffected following incubation with CXCL8–12 (100 ng/mL) for 96 h (*P*>0.05, *n*=6), the same was seen for annexin V^+^/PI^+^ (late apoptotic/necrotic [27]) ASM cells (*P*>0.05, *n*=6) (Figs 4c and d).

Using both DAPI and annexin V/PI staining, no differences were seen in chemokine receptor function between cells derived from asthmatic subjects compared with non-asthmatic controls (data not shown).

## Discussion

We report here for the first time a comprehensive study of chemokine receptor expression by ASM. CCR3, 7, CXCR1, 3 and 4 were highly expressed, but expression was not different between asthmatics and controls. CXCR1, 3 and 4 were functional as evidenced by increased calcium response to ligand activation and promotion of wound healing. However, the effect of recombinant chemokines on wound healing was small and only inhibited by CXCR3 blockade. We have reported previously the ASM expression and function of CCR3 and 7 [21, 22]. In contrast to our earlier findings for CCR3 and 7, activation of CXCR1, 3 and 4 did not mediate ASM migration, but consistent with the CC chemokine receptors, stimulation of the CXC chemokine receptors did not affect ASM proliferation or survival.

Our findings confirm that ASM express CCR1 [19], 3 [20, 21], 7 [22] and CXCR1 [23]. We now extend this panel to include CCR4 and 6, CXCR3, 4 and 6. The chemokine receptors that were highly expressed were CCR3, 7, CXCR1, 3 and 4. Two previous studies have reported that ASM express CCR3, and CCL11 a ligand of CCR3, has the capacity to mediate ASM migration [20, 21]. ASM-derived CCL19, a CCR7 ligand, also mediates ASM migration [22]. We report here for the first time that the CXC chemokine receptors may play a role, albeit minor, in tissue repair in response to injury as assessed by the wound-healing assay. This effect was most marked for CXCR3 as inhibition of this receptor inhibited wound healing in the presence and absence of exogenous chemokines, the latter indicating the involvement of ASM-derived chemokines. Interestingly, we have previously reported that CXCL10 was expressed preferentially by asthmatic ASM in bronchial biopsies and *ex vivo* cells compared with those from healthy control subjects [24]. This supports the view that the CXCL10/CXCR3 axis may play a role in wound repair and maintaining the ASM-bundle integrity. During airway inflammation ASM injury could occur due to the release of various mediators from inflammatory cells and injured epithelial cells, which could result in the expression of various proteins, including chemokines, by ASM [11, 28–30]. Use of the wound-healing assay to mimic the ASM injury, which can occur during inflammation, is validated by the fact that disruption of the ASM monolayer results in the release/expression of a number of cytokines/chemokines that are also released/induced by inflammatory cells [22, 24, 31, 32].

We were unable to demonstrate a chemotactic response of ASM to the chemokines CXCL8–12, suggesting that ASM CXC chemokine receptor expression does not contribute significantly to ASM recruitment. One previous report showed that CXCL8, a ligand for CXCR1, was chemotactic for ASM [23]. It is possible that the discrepancy between our findings and this earlier work and the chemotaxis vs. the wound-healing assays may reflect the relative sensitivity of the assays. However, we have consistently demonstrated that our chemotaxis assay identifies a clear response to PDGF, CCL11 and CCL19, so if our assay is too insensitive to detect a chemotactic response to the CXC chemokines this effect is likely to be very small and therefore of questionable biological importance. Whether the chemokine receptors that were not highly expressed by ASM play a role in ASM migration and wound healing remains unknown and warrants further investigation.

Chemokine receptors, including CXCR1, 3 and 4, have been implicated in the regulation, both positive and negative, of proliferation and survival in a number of cell types [14, 16, 17]. Consequently they can play important roles in processes such as haematopoeisis [15], inflammatory disorders [17, 33] and the progression of cancer [14], and provide potential therapeutic targets [34–36]. Whether chemokine receptors exert an effect on ASM survival or proliferation is uncertain. To date we are only aware of a single report examining this question [21] and in this report CCR3 activation did not affect survival or proliferation. We have extended this observation and using a combination of techniques we have been unable to find a role for CXCL8–12 in either up- or down-regulating ASM proliferation or survival.

Increased ASM mass is a characteristic feature of asthma. The predominant mechanisms driving this ASM hyperplasia are unclear but will be due to increased proliferation, recruitment or prolonged survival of ASM cells either alone or in combination. Our current findings do not support a role for CXCR1, 3 or 4 in ASM migration, proliferation or survival. Earlier work has implicated CCR3 in ASM migration [20, 21]. However, recombinant and ASM-derived CCL11 was inactivated by β-tryptase and co-culture with mast cells [21, 37] and as mast cells are located within the ASM bundle in asthma [24, 38, 39] this questions the importance of ASM CCR3 activation in disease. In contrast to the other chemokine receptors, we have reported that the CCR7/CCL19 axis is important for the migration of ASM cells towards mast cells and the ASM bundle [22]. Importantly, recent evidence also suggests a potential role for ASM progenitor recruitment; ASM progenitors (fibrocytes) are increased in number in the ASM bundle in severe asthma and migration is in part mediated by ASM-derived PDGF [40]. Therefore, although ASM does express a wide panel of chemokine receptors, their potential function in modulating ASM hyperplasia appears to be limited, with CXCR3 and CCR7 mediating wound repair and CCR7 promoting ASM migration.

One potential criticism of our study is that we may have underestimated the importance of chemokine receptor function in ASM as we have not examined the effects following priming with pro-inflammatory cytokines, which play a role in disease, or the combined effects of several chemokines. However, we have shown that ASM chemokine receptor mRNA expression is not different between ASM from asthmatic and non-asthmatic subjects and is unaffected following incubation with poly(I:C) or HLMC lysates which mimic viral infection and the inflammatory milieu, respectively. Additionally, we have previously examined the effect of mast cell–ASM interactions on ASM migration and this has highlighted the importance of the CCL19/CCR7 axis only [22]. We are therefore confident that it is unlikely that we have overlooked any biologically important effects of the other chemokine receptors expressed by ASM in the pathogenesis of asthma.

In conclusion, we have described the panel of chemokine receptors expressed by ASM from subjects with and without asthma. CCR3, 7, CXCR1, 3 and 4 were the most highly expressed receptors. Expression was not different between health and disease. Further study of the function of CXC chemokine receptors revealed that they are functional and mediate wound repair but not migration, proliferation or survival. This suggests that these chemokine receptors may modulate some aspects of ASM function, but does not support a key role for CXCR1, 3 or 4 in the development of ASM hyperplasia in asthma.
